# Delayed Fluoxetine Administration Restores Hippocampal Function in a Juvenile Global Cerebral Ischemia Mouse Model in a Sex‐Specific Manner

**DOI:** 10.1155/np/8841616

**Published:** 2025-12-19

**Authors:** April Fineberg, Tanner McVey, Jamie Henry, Erika Tiemeyer, James E. Orfila, Robert M. Dietz

**Affiliations:** ^1^ Department of Pediatrics, University of Colorado, Anschutz Campus, Aurora, Colorado, USA, colorado.edu; ^2^ Department of Neurological Surgery, The Ohio State University College of Medicine, Columbus, Ohio, USA, osu.edu

**Keywords:** cardiac arrest, fluoxetine, global cerebral ischemia, juvenile, pediatric

## Abstract

Global cerebral ischemia (GCI) during childhood is a leading cause of long‐term cognitive impairment, yet no therapies currently exist to promote recovery in survivors. We previously demonstrated that juvenile mice exhibit transient hippocampal synaptic dysfunction after GCI, associated with reduced brain‐derived neurotrophic factor (BDNF) expression and partial endogenous recovery over time. In this study, we tested whether delayed treatment with fluoxetine (FLX)—a selective serotonin reuptake inhibitor (SSRI) known to enhance BDNF–TrkB signaling—could accelerate synaptic recovery. Juvenile mice underwent cardiac arrest and cardiopulmonary resuscitation, followed by in vivo FLX or vehicle administration from postinjury days 10–13. Electrophysiological recordings on day 14 revealed that FLX restored hippocampal long‐term potentiation (LTP) in males but not females. This effect was paralleled by an increase in hippocampal BDNF expression in FLX‐treated males, whereas no change was observed in females. Paired ex vivo experiments further confirmed that acute FLX exposure rescued LTP in GCI‐injured male slices. These findings suggest that FLX promotes synaptic recovery through BDNF–TrkB signaling in males, while recovery in females may proceed via alternate, hormone‐dependent mechanisms. Together, these results identify a novel therapeutic window for enhancing neuroplasticity after juvenile GCI and underscore the importance of developmental stage and biological sex in shaping responses to treatment.

## 1. Introduction

Cardiac arrest remains a leading cause of global cerebral ischemia (GCI) across all age groups. Although survival rates have improved due to advances in resuscitation techniques, the long‐term neurological consequences remain severe, particularly in developing brains [1–7]. In children, over 20,000 cases of cardiac arrest occur annually in the United States, often due to respiratory failure, trauma, or arrhythmia [8]. Survival rates in children vary dramatically based on setting, with in‐hospital arrests associated with ~60% survival compared to only ~19% for out‐of‐hospital events [8]. Survivors frequently experience persistent cognitive deficits, notably in memory and learning, which can affect developmental milestones and academic achievement [1–3, 5]. Some require long‐term support due to significant motor and cognitive disabilities [1, 2]. While therapeutic hypothermia is neuroprotective in neonates and adults [9, 10], randomized trials in pediatric populations have not demonstrated the same benefit [11, 12]. These discrepancies underscore the need to elucidate age‐specific mechanisms of injury and recovery to inform pediatric‐focused interventions.

Brain‐derived neurotrophic factor (BDNF) plays a critical role in neuronal survival, synaptic plasticity, and cognitive function [13–18]. We previously reported that BDNF expression transiently decreases in the hippocampus after juvenile GCI, correlating with impaired long‐term potentiation (LTP)—a widely accepted surrogate for memory formation [14]. Although BDNF has been shown to be neuroprotective in neonatal hypoxic‐ischemic injury [19], it has not improved outcomes in adult models of cerebral ischemia [20], reinforcing the importance of age‐dependent mechanisms [3]. This developmental divergence likely reflects fundamental differences in synaptic architecture, neurotrophin signaling, and hormonal milieu between immature and mature brains. For example, BDNF expression and TrkB receptor isoform distribution vary across early postnatal development, influencing how plasticity‐related pathways respond to injury [21, 22]. In addition, the juvenile brain exhibits a greater proportion of silent synapses, heightened activity‐dependent plasticity, and a delayed hormonal environment, all of which may alter its capacity for neurorestoration compared to adults [3, 23, 24]. Notably, we have demonstrated that pharmacologic activation of TrkB, the high‐affinity receptor for BDNF, is sufficient to restore hippocampal synaptic function in juvenile mice following GCI, providing direct evidence that the developing brain remains responsive to targeted neurotrophic signaling during a delayed recovery window [14].

Building on this work, the present study investigates whether fluoxetine (FLX), a selective serotonin reuptake inhibitor (SSRI) widely prescribed in children and adolescents [25, 26] can similarly restore synaptic plasticity after juvenile GCI, offering a clinically translatable approach to engage the same signaling axis. Although best known for its antidepressant effects via serotonergic modulation [27], FLX also enhances BDNF expression and TrkB signaling in preclinical models [28–31], along with additional nonserotonergic effects [32]. Given our interest in the recovery of synaptic function after juvenile brain injury, we used hippocampal LTP and memory behavior as functional readouts to assess the effect of delayed FLX administration. While LTP is a well‐established model of hippocampal‐dependent plasticity and memory, prior studies report inconsistent effects of FLX: some show no change in LTP [33, 34], others describe impairment following chronic exposure [35], and still others report enhanced potentiation via a BDNF‐dependent mechanism [36]. These discrepancies likely reflect differences in experimental context, including the age of animals, duration and timing of FLX administration, and presence or absence of prior injury. Notably, most studies have focused on adult animals or noninjured models, whereas our approach targets a distinct developmental window in the juvenile brain; a window that we have previously shown is marked by impaired synaptic plasticity and reduced BDNF expression following GCI [3, 14]. Furthermore, FLX failed to improve outcomes in recent clinical trials in adult stroke patients [37–39], raising questions about its therapeutic potential in mature ischemic brain injury. By contrast, the juvenile brain retains a unique capacity for recovery [14] and may be more responsive to neurorestorative interventions. Our model is thus uniquely positioned to test the efficacy of FLX in a clinically relevant setting that accounts for both developmental stage and injury context.

Here, we demonstrate that delayed FLX administration restores hippocampal synaptic plasticity in juvenile mice following GCI in a sex‐specific manner, likely through upregulation of BDNF. Mice at postnatal day (P) 21–25, a neurodevelopmental stage estimated to approximate that of a 3‐ to 4‐year‐old child [24], underwent cardiac arrest using established methods [14, 40–42]. Hippocampal function was assessed 14 days later, corresponding to an age approximating early adolescence in humans (12–14 years) [24]. We selected a delayed treatment window based on our prior work demonstrating that hippocampal synaptic plasticity can be restored well after the acute reperfusion period [14, 42]. This juvenile window represents a critical developmental period. We have previously identified impaired synaptic plasticity following cardiac arrest, associated with reduced hippocampal BDNF expression [14]. These findings reveal a previously unrecognized therapeutic window in the developing brain and support the potential for FLX repurposing to enhance cognitive recovery in children after cardiac arrest. More broadly, this work highlights the critical role of developmental stage and biological sex in shaping therapeutic efficacy and underscores the need for age‐specific strategies in pediatric neurocritical care.

## 2. Methods

### 2.1. Experimental Animals

All experimental protocols were approved by the University of Colorado‐Denver Institutional Animal Care and Use Committee (IACUC) and conformed to the National Institutes of Health guidelines for care and use of animals. Male and female C57Bl/6 20–25 day old (PND 20–25, prepubertal) mice (Charles River Laboratory) were used for this study. These mice were weaned and not with dam at the time of experiment. The mice were housed in a standard 12 h light and 12 h dark cycle and had free access to food and water. All experiments in the study adhered to the ARRIVE 2.0 guidelines for animal experiments [43]. Mice were randomly assigned to experimental groups, and the investigator was blinded through analysis.

### 2.2. Cardiac Arrest and Cardiopulmonary Resuscitation

Cardiac arrest with resuscitation was performed in juvenile mice (P21–25) following previously validated protocols [14, 41, 42, 44], with updates for clarity and reproducibility. Mice were anesthetized using 3% isoflurane and maintained with 2%–2.5% isoflurane in 21% fraction of inspired oxygen (FiO_2_) via a face‐mask. Body temperature was closely monitored and kept near at 37°C using both a heating pad and a heat lamp with thermistors placed in the rectum and left ear canal. For intravenous access, a PE‐10 catheter was inserted into the right internal jugular vein through a small neck incision and flushed with heparinized saline. Endotracheal intubation was achieved using a 24G catheter, followed by mechanical ventilation (Minivent; Hugo Sachs Elektronik, Germany) at a rate of 160 breaths per minute. Cardiac activity was tracked throughout via surface electrocardiogram (ECG). To induce cardiac arrest, anesthesia was discontinued 1 min before the procedure. Arrest was initiated by intravenous injection of 30 µL of 0.5 M KCl through the jugular catheter, immediately followed by cessation of mechanical ventilation. Asystole was confirmed by ECG and the absence of spontaneous breathing. Heating was stopped 1 min before arrest, and during the arrest period, pericranial temperature was held at 37.5 ± 0.5°C using a water‐cooled coil or heat lamp, while core body temperature was allowed to fall passively to ~35°C. Thirty seconds prior to compressions, ventilation resumed with 100% oxygen at 210 breaths/min. At 8 min, chest compressions (~300/min) were initiated, accompanied by a slow IV injection (0.2–0.5 mL) of an epinephrine/calcium chloride solution (16 µg/mL each in 0.9% saline). Resuscitation continued until return of spontaneous circulation (ROSC), defined by ECG evidence of organized electrical activity. Mice not achieving ROSC within 3 min were excluded. Following resuscitation, oxygen concentration was reduced to 50%, and weaning from ventilation proceeded in stages as spontaneous breathing increased. Once respiratory rates exceeded 60 breaths/min, the endotracheal tube was removed. Catheters and probes were withdrawn, and mice were transferred to individual cages for recovery. Experimental group allocation and downstream analyses were performed blinded to condition.

### 2.3. Drug Treatment

Male and female mice were injected via retroorbital route with FLX hydrochloride (15 mg/kg, Thermo Scientific, Waltham, MA) or vehicle on Days 10, 11, 12, and 13 after GCI or sham surgeries and studied on Day 14. In a separate set of paired experiments, acute hippocampal slices were collected on Day 14 after GCI or sham surgery and subjected to vehicle or FLX (5 *μ* M) for 2–4 h prior to electrophysiology experiments.

### 2.4. Acute Hippocampal Slice Preparation

Hippocampal slices were prepared at 14 days cardiac arrest or sham procedures. Mice were anesthetized in an oxygen‐enriched induction chamber using 3.5%–4% isoflurane. To rapidly reduce brain temperature and improve tissue preservation, animals were perfused transcardially with ice‐cold, carbogenated (95% O_2_/5% CO_2_) artificial cerebrospinal fluid (aCSF) for approximately 2 min prior to decapitation. Brains were then swiftly removed and submerged in the same oxygenated aCSF. The composition of aCSF was the following (in mmol/L): 126 NaCl, 2.5 KCl, 25 NaHCO_3_, 1.3 NaH_2_PO_4_, 2.5 CaCl_2_, 1.2 MgCl_2_, and 12 glucose [45]. Horizontal hippocampal sections (300 *μ* m) were generated using a Vibratome 1200 (Leica Microsystems). Slices were transferred to a submerged holding chamber containing oxygenated aCSF and allowed to recover for at least 1 h at room temperature before being used in electrophysiological recordings.

### 2.5. Electrophysiology

Field excitatory postsynaptic potentials (fEPSPs) were recorded from the CA1 region of acute hippocampal slices maintained in a temperature‐controlled interface recording chamber (31 ± 0.5°C), continuously perfused with oxygenated aCSF at a flow rate of 1.5 mL/min. Schaffer collateral axons were stimulated to evoke fEPSPs, which were recorded in the stratum radiatum. Stimulus intensity was adjusted to evoke a response with a slope at 50% of maximum, and test pulses were delivered every 20 s to assess baseline synaptic transmission. Paired pulse responses were recorded using a 50 ms interpulse interval (20 Hz) and expressed as a ratio of the slopes of the second pulse over the first pulse. After establishing a 20 min stable baseline, LTP was induced using a theta burst stimulation (TBS) train of four pulses delivered at 100 Hz in 30 ms bursts repeated 10 times with 200 ms interburst intervals [45]. Synaptic potentiation was monitored for 60 min following TBS, and the average slope of fEPSPs recorded during minutes 50–60 was normalized to the pre‐TBS baseline (set to 100%) to quantify LTP magnitude. Analog signals were amplified (1000×) and filtered through a pre‐amplifier (Model LWE has 11 AC, Grass Instruments) at 1.0 kHz, digitized at 10 kHz, and stored on a computer for later offline analysis (Clampfit 10.7, Axon Instruments). The initial slope of the fEPSP was quantified as the first derivative (*dV*/*dT*). Time‐course data were plotted as percent change from baseline. Pre‐established exclusion criteria included removal of slices in which control LTP was less than 120% of baseline.

### 2.6. Protein Fractionation

A bullet blender was used to homogenize hippocampal tissue in neuronal protein extraction reagent (NPER) buffer containing protease and phosphatase inhibitors and 0.5 mm zirconium oxide beads. Protein concentration was measured with a Pierce BCA Protein Assay Kit (ThermoFisher Scientific). Protein lysates were stored at −80°C until time of experiment.

### 2.7. ELISA

Protein extracts were collected, and BDNF levels were measured using a Mouse BDNF ELISA kit (LS‐F2404; LifeSpan Biosciences, Seattle, WA, USA) as per the manufacture’s instructions. Samples were diluted using the sample diluent from the kit, then loaded onto a plate and incubated for 90 min at 37°C. After aspirating liquid from each well, 1x detection antibody solution was added and left to incubate for 1 h at 37°C. After washing 3x, 1xABC Complex working solution was added and left to incubate for 30 min at 37°C. After washing 5x, TMB Substrate was added and left to incubate for 20 min at 37°C. Stop solution was added, and optical density was measured at 450 nm immediately using the SpectraMax M5 microplate reader (Molecular Devices, Sunnyvale, CA, USA). All BDNF concentration values were normalized to total protein content in the sample measured with a Pierce BCA Protein Assay Kit (ThermoFisher Scientific).

### 2.8. Western Blot

Protein samples (5–6 µg) were separated on 4%–20% Mini‐PROTEAN TGX Precast Protein Gels and electro‐transferred to 0.2 µm polyvinylidene difluoride (PVDF) membranes utilizing Trans‐Blot Turbo Transfer system (Bio‐Rad, 1704150) by Bio‐Rad Laboratories. PVDF membranes were blocked in 5% bovine serum albumin (BSA) or 8% milk for 1 h and incubated overnight at 4°C in primary antibody. The following primary antibodies were used: phospho‐TrkB (Tyr816) Polyclonal Antibody (1:1000; phospho‐TrkB, Osenses, catalog #OST00220W), phospho‐TrkB (Tyr705) Polyclonal Antibody (1:1000; Invitrogen, catalog #PA5‐105012), phospho‐TrkB (Tyr‐516) Polyclonal Antibody (1:1000; Invitrogen, catalog #PA5‐36695). All membranes were washed three times and incubated in donkey anti‐rabbit Horseradish peroxidase conjugated antibody (1:10,000, Thermo Scientific) for 1 h at room temperature. After three washes, bands were detected using SuperSignal West Femto Maximum Sensitivity Substrate (ThermoScientific, 34096) and imaged with ChemiDocTM MP Imaging System (Bio‐Rad, Hercules, CA, USA). Phospho‐TrkB expression was normalized to mouse anti‐*β* actin peroxidase (1:5000, A3854; Sigma). All membranes were washed three times and incubated in TrkB Polyclonal Antibody (1:1000 Osenses, catalog #OST00336W) overnight at 4°C. The membranes were washed three times and then incubated in donkey anti‐goat IgG Secondary Horseradish peroxidase conjugated antibody (Invitrogen, catalog #A15999) for 1 h at room temperature. After three washes, the western blot bands were detected using SuperSignal West Pico PLUS Chemiluminescent Substrate (ThermoFisher Scientific, 34580). Quantification of the integrated volume of bands was performed using IMAGE LAB software version 4.0 (Bio‐Rad). Protein expression was quantified as a ratio of normalized phospho‐TrkB to total TrkB.

### 2.9. Statistical Analysis

All analyses were conducted in a blinded fashion, with the investigator performing statistical assessments distinct from the individual conducting the experimental recordings. Data are reported as mean ± standard error of the mean (SEM). Prior to experimentation, power calculations were performed using 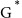
Power software. Based on prior observations, detecting a 40% difference in LTP magnitude between experimental groups (assuming a standard deviation of 15%, *α* = 0.05, *β* = 0.20) required a minimum of four slices per condition to achieve 80% statistical power. To minimize the impact of intra‐animal variability, no more than two slices per animal were included in each condition. Statistical comparisons between groups were conducted using one‐way analysis of variance (ANOVA), followed by post hoc Dunnett’s test for comparisons against a single control group. A *p*‐value of less than 0.05 was considered statistically significant and is indicated in figures by an asterisk.

## 3. Results

### 3.1. Delayed Administration of FLX Recovers LTP Impairment Following GCI

Juvenile mice (P 21–25) were subjected to either sham procedures or GCI induced by 8 min of asystolic cardiac arrest, followed by resuscitation, using protocols previously established in our laboratory [14, 41, 42]. To evaluate hippocampal synaptic plasticity, LTP was quantified by calculating the percent change in the slope of fEPSPs relative to the pre‐stimulation baseline (normalized to 100%). In slices from sham‐operated mice 14 days postsurgery, TBS (40 pulses at 100 Hz) induced robust LTP, with fEPSP slopes reaching 166% ± 14% of baseline after 60 min (*n* = 4, Figure 1). Synaptic potentiation was significantly reduced following GCI (107% ± 4.2%; *n* = 4, *p*  < 0.05 vs. sham, Figure 1). Remarkably, in paired ex vivo experiments, hippocampal slices from GCI‐injured mice treated with FLX (5 *μ* M) for 3–4 h prior to and during recording exhibited restored LTP, reaching 148% ± 12% of baseline (*n* = 4, *p*  < 0.05 vs. GCI + vehicle, Figure 1). FLX had no significant effect on LTP in sham‐operated slices (Figure 1), indicating that its restorative effect is specific to the injured state. These results demonstrate that ex vivo FLX treatment can rescue synaptic plasticity following juvenile GCI.

Figure 1Ex vivo delayed administration of fluoxetine restores synaptic function. (A) Timeline of experimental design. (B) LTP time plot of fluoxetine effect on sham slices. (C) Quantification of LTP from sham slices. (D) LTP time plot of fluoxetine effect on GCI slices. (E) Quantification of LTP from GCI slices. Each point indicates an individual experiment; no more than two experiments per animal. Black points indicate vehicle treatment, red points indicate FLX treatment.  ^∗^ *p*  < 0.05.

**Figure:**
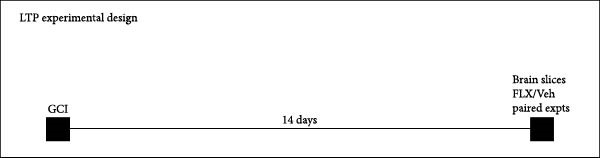
(A)

**Figure:**
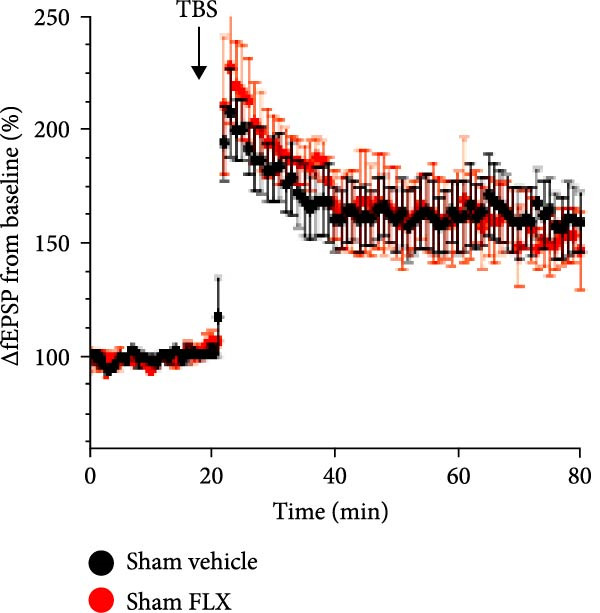
(B)

**Figure:**
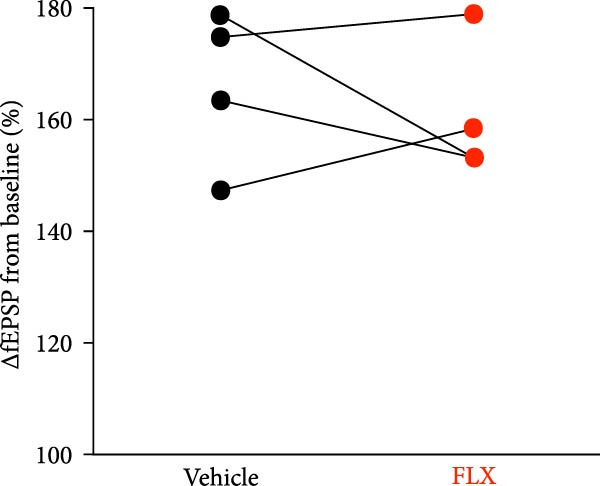
(C)

**Figure:**
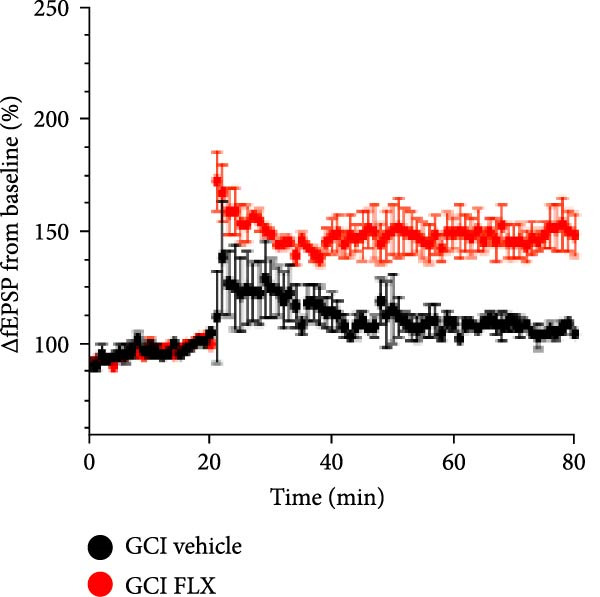
(D)

**Figure:**
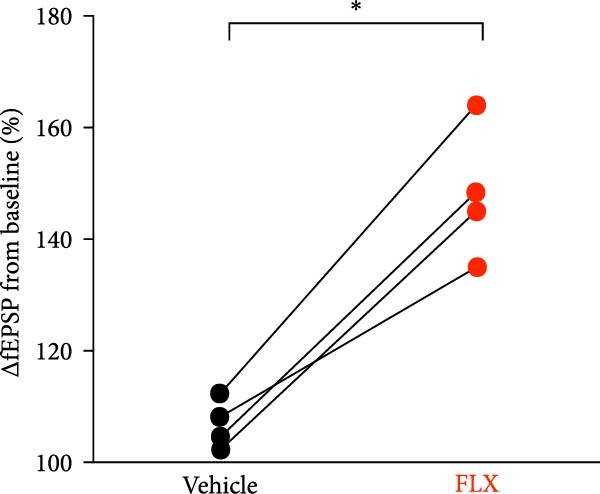
(E)

We next tested whether in vivo FLX treatment could restore hippocampal synaptic plasticity following GCI. Male and female juvenile mice underwent either sham or GCI surgeries and received FLX (15 mg/kg) or vehicle once daily on postinjury days 10–13 (Figure 2A). On Day 14, acute hippocampal slices were prepared, and LTP was assessed. In both sexes, GCI significantly impaired LTP compared to sham controls. In males, LTP was reduced from 187% ± 31% in shams to 114% ± 22% following GCI (*n* = 6 per group, *p*  < 0.05, Figure 2B,C). Similarly, females exhibited reduced LTP after GCI (151% ± 15% in shams vs. 110% ± 11% after GCI; *n* = 6–7, *p*  < 0.05, Figure 2D,E). In vivo FLX treatment had no effect on LTP in sham‐operated animals of either sex, indicating no baseline enhancement of synaptic plasticity. However, in GCI‐injured males, FLX significantly restored LTP compared to vehicle‐treated controls (163% ± 35% vs. 114% ± 22%, *n* = 6 per group, *p*  < 0.05, Figure 2C). In contrast, FLX had no effect in GCI‐injured females (117% ± 31% vs. 110% ± 11%, *n* = 6 per group, *p* = 0.95). These results confirm that delayed in vivo FLX treatment promotes recovery of synaptic plasticity following juvenile GCI in a sex‐specific manner.

Figure 2In vivo administration of fluoxetine rescues LTP in a sex‐specific manner. (A) Timeline of experimental design showing drug or vehicle injection on Days 10, 11, 12, and 13 after surgery. (B) Time plot of LTP in male juvenile mice. (C) Quantification of LTP in sham and GCI male mice treated with vehicle (Veh) or fluoxetine (FLX). (D) Time plot of LTP in female juvenile mice. (E) Quantification of LTP in sham and GCI female mice treated with vehicle (Veh) or fluoxetine (FLX). Each point indicates an individual experiment; no more than two experiments per animal. Black points indicate vehicle treatment, red points indicate FLX treatment, circles indicate sham surgery, and triangles indicate GCI surgery.  ^∗^ *p*  < 0.05,  ^∗∗^ *p*  < 0.01.

**Figure:**
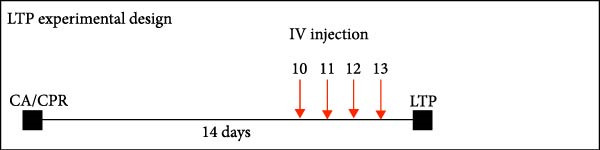
(A)

**Figure:**
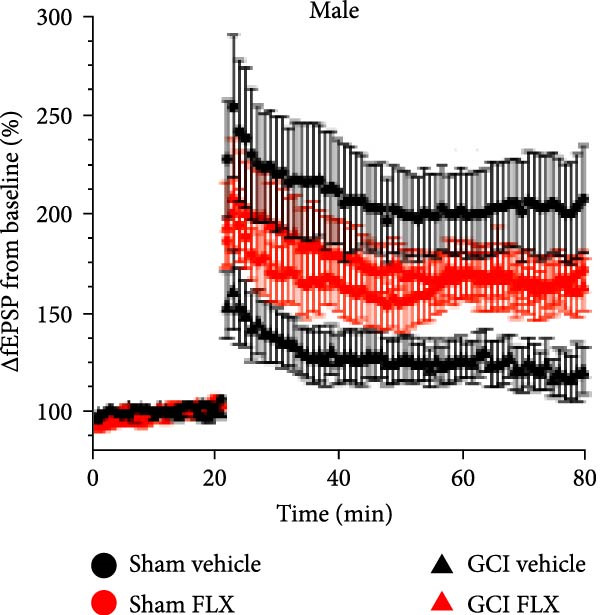
(B)

**Figure:**
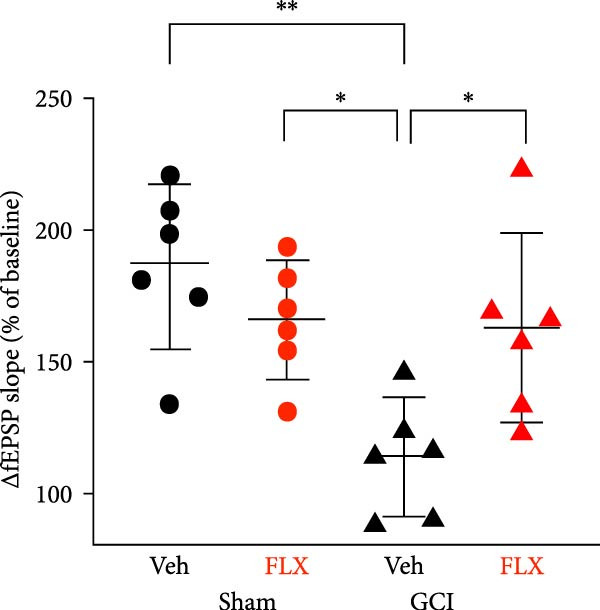
(C)

**Figure:**
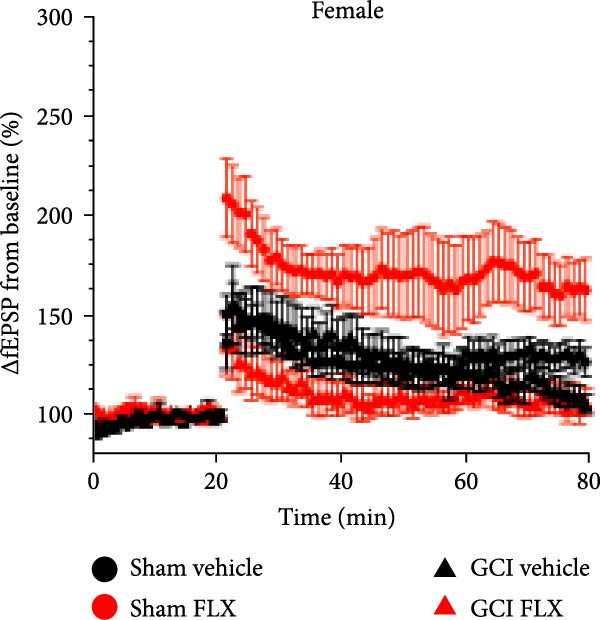
(D)

**Figure:**
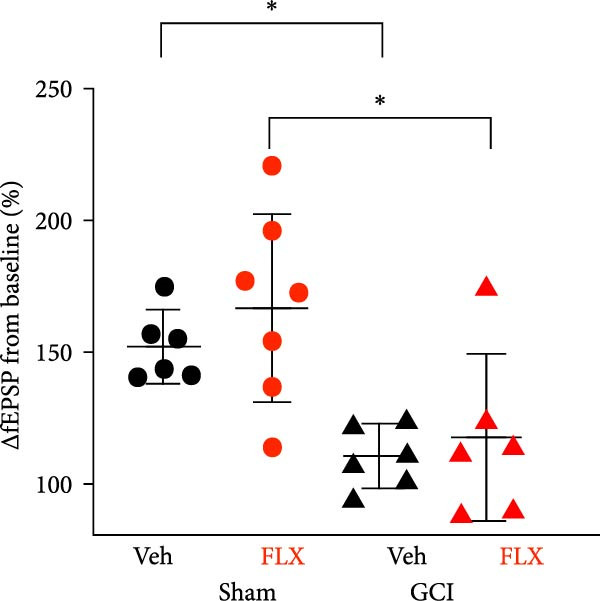
(E)

### 3.2. In Vivo FLX Administration Increases BDNF Expression

FLX has been previously shown to increase BDNF expression in multiple brain regions [46–48], prompting the hypothesis that its restorative effect on LTP may be mediated by enhanced endogenous BDNF signaling. To test this, hippocampal slices from male mice were treated ex vivo with vehicle or FLX (5 *μ* M) for 3–4 h, and BDNF protein levels were measured by ELISA. Consistent with our prior findings [14], GCI reduced BDNF expression in vehicle‐treated slices compared to sham controls (*p*  < 0.05, Figure 3A). However, FLX treatment significantly increased BDNF levels in GCI‐injured slices (*p*  < 0.01 vs. GCI + vehicle, Figure 3A), suggesting that FLX restores synaptic plasticity by reversing the injury‐induced loss of BDNF.

Figure 3Effect of fluoxetine on BDNF expression measured by ELISA. (A) BDNF expression following a single ex vivo dose of fluoxetine on Day 14 following surgery in male mice showing decreased BDNF in the vehicle‐treated GCI group, contrasted by an increase in BDNF expression following treatment with fluoxetine in the GCI group. (B) BDNF expression following in vivo administration of fluoxetine on Days 10, 11, 12, and 13, followed by hippocampal isolation on Day 14 in male mice. (C) BDNF expression following in vivo administration of fluoxetine on Days 10, 11, 12, and 13, followed by hippocampal isolation on Day 14 in female mice. Each point indicates an individual animal, black points indicate vehicle treatment, red indicates FLX treatment, circles indicate sham surgery, and triangles indicate GCI surgery.  ^∗^ *p*  < 0.05,  ^∗∗^ *p*  < 0.01,  ^∗∗∗∗^ *p*  < 0.001.

**Figure:**
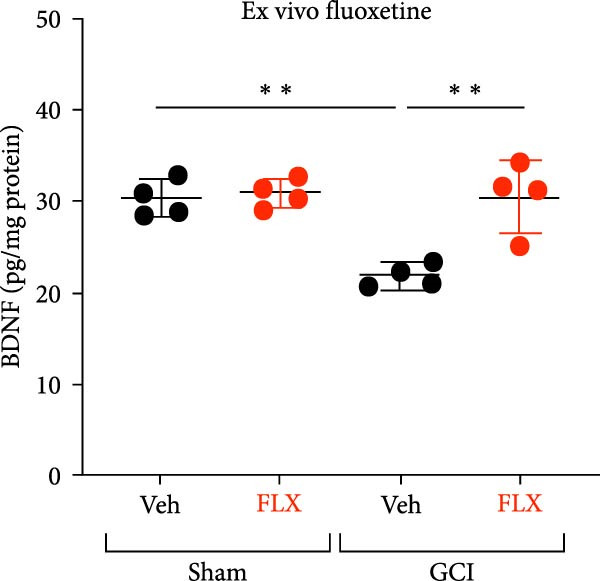
(A)

**Figure:**
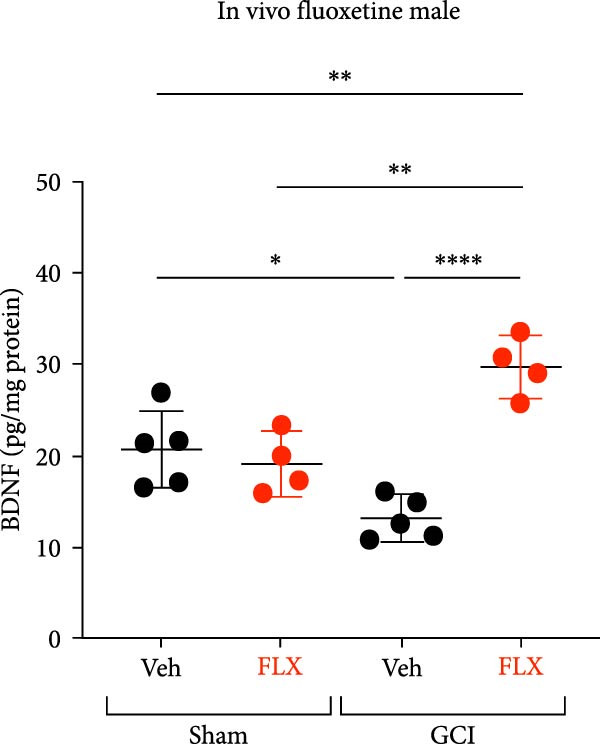
(B)

**Figure:**
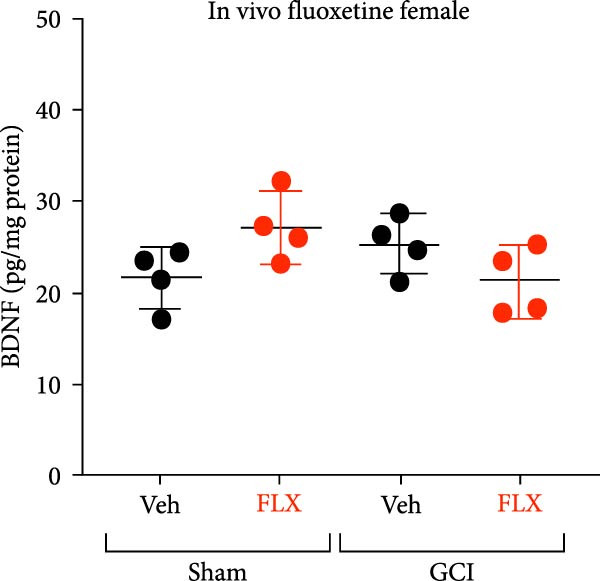
(C)

To determine whether this effect also occurs in vivo, a separate cohort of juvenile mice received FLX (15 mg/kg) or vehicle once daily on postinjury days 10–13. Whole hippocampi were collected on Day 14, and BDNF levels were quantified. In male mice, FLX treatment significantly increased hippocampal BDNF expression in the GCI group compared to GCI + vehicle and both sham groups (*p*  < 0.01, Figure 3B). In contrast, BDNF expression in females did not differ across surgical or treatment conditions (Figure 3C). These results indicate that delayed, repeated in vivo administration of FLX enhances BDNF expression selectively in males, supporting its role in mediating the observed recovery of synaptic plasticity following juvenile GCI.

### 3.3. FLX Does Not Alter Activation of Trk‐B Receptors

Recent evidence suggests that FLX can directly bind and activate Trk‐B receptors [28], raising the possibility that FLX may act independently of BDNF to promote synaptic recovery. TrkB activation involves phosphorylation at multiple tyrosine residues, each mediating distinct intracellular signaling cascades relevant to synaptic plasticity [49–53]. Phosphorylation of tyrosine 816 enables recruitment of phospholipase C‐*γ*1 (PLC*γ*1), triggering calcium mobilization and protein kinase C activation—both critical for LTP and learning. Tyrosine 516 serves as a docking site for adaptor proteins such as Shc and FRS2, facilitating downstream Ras/MAPK and PI3K/Akt signaling involved in neuronal survival and differentiation. Meanwhile, phosphorylation of tyrosine 705, located within the TrkB kinase activation loop, is required for full catalytic activation of the receptor and subsequent autophosphorylation events.

**Figure 4:**
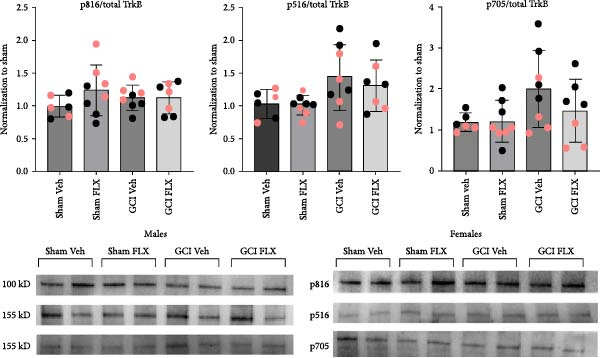
TrkB phosphorylation does not change following GCI or fluoxetine treatment. Phosphorylation of serine residues 816, 516, and 705 was evaluated in whole hippocampus from male and female juvenile mice following in vivo administration of vehicle or fluoxetine on Days 10, 11, 12, and 13, followed by tissue collection on Day 14. All ratios were normalized to the sham vehicle group. Black dots indicate data from males, and pink dots indicate data from females.

To determine whether FLX enhances synaptic function via direct TrkB activation, we collected whole hippocampi from male and female mice 14 days postsurgery, following in vivo treatment with FLX (15 mg/kg) or vehicle on Days 10–13. Immunoblot analysis was performed to assess phosphorylation at TrkB tyrosines 705, 516, and 816 relative to total TrkB levels. As shown in Figure 4, FLX treatment did not alter phosphorylation of any of these residues in either sex, suggesting that FLX does not act through direct activation of TrkB to promote synaptic recovery following GCI. These findings support the hypothesis that FLX enhances synaptic plasticity through upregulation of endogenous BDNF rather than direct receptor engagement.

## 4. Discussion

In this study, we investigated whether delayed administration of FLX, an SSRI known to increase BDNF levels, could promote recovery of synaptic plasticity following juvenile GCI. Our results demonstrate that FLX restored hippocampal LTP in juvenile male mice when administered 10–13 days after GCI, indicating that synaptic function in the developing brain remains responsive to intervention well beyond the acute injury phase. GCI in early life is associated with persistent cognitive impairments, particularly in learning and memory domains [3], yet current treatment options remain limited for supporting functional recovery in pediatric survivors. Extending our previous findings that juvenile GCI induces a transient disruption of hippocampal LTP, accompanied by downregulation of BDNF and gradual spontaneous recovery over time [14], the present data support the hypothesis that pharmacologically enhancing BDNF signaling can accelerate or amplify this endogenous repair process. The effectiveness of FLX in rescuing synaptic plasticity within this delayed treatment window underscores its potential as a neurorestorative agent and highlights the importance of timing and developmental context when designing postischemic interventions.

Mechanistically, we found that in vivo FLX administration restored hippocampal BDNF expression in male mice following GCI, corresponding with the recovery of LTP. In contrast, female mice showed no increase in BDNF levels and no improvement in synaptic plasticity following FLX administration, indicating a sex‐specific response to FLX. These findings support the idea that BDNF–TrkB signaling is essential for FLX‐mediated restoration of synaptic function in males. Treatment was initiated several days after the ischemic event (postinjury days 10–13) to align with our prior findings that deficits in synaptic plasticity in juvenile mice remain reversible well beyond the acute reperfusion period [14]. By selecting this later window, our goal was to evaluate FLX as a neurorestorative intervention rather than an acute neuroprotective therapy, thereby modeling a clinically relevant timeframe in which children typically present for ongoing care after cardiac arrest. The dosing and relatively short duration of FLX exposure (15 mg/kg for 4 days) were selected to align with prior preclinical studies, while maintaining the emphasis on delayed intervention. Although it is possible that earlier or prolonged treatment could yield different outcomes, particularly in females, the goal of this study was to test whether a brief, delayed therapeutic window could restore synaptic function during this juvenile stage. This effect was further validated in paired *ex vivo* experiments, where brief FLX exposure was sufficient to rescue LTP in hippocampal slices from male GCI mice. The rapid onset of this response aligns with previous reports that FLX can enhance BDNF–TrkB activity within minutes [54]. Although the antidepressant effects of FLX are typically observed only after prolonged treatment, our data suggest that its influence on synaptic plasticity may occur on a much shorter timescale.

To determine whether FLX directly activates TrkB receptors, as suggested in recent studies [28], we assessed TrkB phosphorylation following a 4‐day FLX regimen. However, we observed no significant changes in phosphorylation at key TrkB tyrosine residues (Y705, Y516, Y816), suggesting that increased BDNF availability rather than sustained receptor activation mediates its effects on synaptic function. This apparent disconnect does not exclude a role for TrkB signaling; receptor phosphorylation can be highly transient and may return to baseline before tissue harvest, or downstream effectors such as PI3K/Akt, MAPK/ERK, or CREB may remain activated independently of detectable receptor phosphorylation [55]. Moreover, FLX has been reported to influence BDNF signaling indirectly, for example, by modifying TrkB localization within membrane microdomains or facilitating receptor–effector interactions without robust changes in phosphorylation [28]. These alternative mechanisms may explain how enhanced BDNF expression promotes synaptic recovery despite unchanged steady‐state TrkB phosphorylation. These findings highlight the importance of considering not only receptor activation, but also downstream and membrane‐associated signaling mechanisms when designing BDNF‐targeted therapies for pediatric brain injury.

Intriguingly, our findings indicate that recovery mechanisms in juvenile female mice following GCI differ from those in males and do not appear to depend on BDNF signaling. This is consistent with our recent work [56], which demonstrated that LTP recovery in juvenile females after GCI is delayed and instead mediated by estrogen receptor alpha (ER*α*) activation, independent of BDNF upregulation. Although we did not assess estrous cycle stage in this study, the timing of hormonal surge during puberty onset in female mice (P 56–62) [57] is well beyond the age of animals used here, making it unlikely that fluctuating sex hormones or cycle‐related variability contributed significantly to the observed effects. Nonetheless, the absence of FLX‐mediated BDNF induction or LTP rescue in females highlights a sexually dimorphic response in the juvenile brain. Future studies will be needed to determine how delayed hormonal maturation and ER*α* signaling interact with neurorestorative mechanisms during this critical window. Together, these findings underscore the importance of developmental stage and sex‐specific molecular pathways in shaping recovery after brain injury and support the need for targeted therapeutic strategies that account for biological sex.

Our findings reinforce the emerging concept that neurorestorative strategies may offer a valuable therapeutic window following pediatric GCI [3]. The restoration of hippocampal LTP by FLX, administered nearly 2 weeks after injury, suggests that synaptic dysfunction in surviving neurons remains amenable to modulation well beyond the acute postischemic period. Importantly, this recovery coincides with a developmental stage during which children are particularly vulnerable to disruptions in learning and memory processes. Given that spontaneous recovery of hippocampal function occurs by 30 days post‐GCI in juvenile mice, enhancing synaptic function during this window may accelerate neurodevelopmental repair and mitigate long‐term cognitive deficits. The use of FLX, a widely prescribed and FDA‐approved antidepressant for pediatric populations [25, 26], adds translational relevance and feasibility to this approach. These data support a shift in therapeutic focus from early neuroprotection to delayed intervention targeting network reorganization and synaptic remodeling as a promising avenue to improve functional outcomes after childhood cardiac arrest. Importantly, our findings may shed light on the lack of efficacy observed in recent clinical trials evaluating FLX as a neurorestorative therapy in adult stroke patients [37–39]. The failure of these trials may stem from fundamental age‐dependent differences in the mechanisms of injury and recovery. Whereas adult brains exhibit sustained impairments in synaptic plasticity and limited capacity for regeneration following GCI [45, 58], the juvenile brain retains a higher degree of plasticity [3, 14, 41, 42], potentially making it more amenable to restorative interventions. Our data demonstrate that FLX can effectively rescue LTP in juvenile male mice, suggesting that delayed activation of BDNF signaling may be a viable therapeutic strategy in pediatric populations. However, the observed sex‐specific response highlights the necessity of considering developmental and hormonal context in the design of age‐appropriate therapies for ischemic brain injury.

Despite these promising findings, important questions remain. The molecular basis underlying the sex‐specific response to FLX is not yet fully understood and warrants further investigation. Prior studies from our group suggest that ER*α* signaling plays a key role in synaptic recovery in females [56], potentially bypassing or modulating the BDNF–TrkB pathway. Whether FLX can interact with these hormone‐dependent mechanisms, or whether combination therapies targeting both neurotrophic and hormonal pathways may be required to achieve recovery in females, remains an open and compelling area of inquiry. Moreover, while LTP serves as a robust cellular correlate of memory and often correlates with memory behavior [14, 41, 42, 56, 58–61], future studies incorporating behavioral assays will be critical to determine whether FLX‐mediated restoration of synaptic plasticity translates to improved cognitive outcomes following juvenile GCI.

## 5. Conclusion

In summary, our findings demonstrate that delayed FLX treatment can restore hippocampal synaptic plasticity after juvenile GCI in a sex‐specific manner, likely through BDNF–TrkB signaling in males. These results reveal a previously underappreciated therapeutic window in the developing brain during which synaptic function remains amenable to intervention, even well after the acute injury phase. By leveraging this period of heightened plasticity, FLX or related modulators of BDNF signaling could represent promising adjunctive strategies to enhance neurorestorative care in pediatric patients following cardiac arrest or GCI. The observed sex differences highlight the importance of incorporating biological sex and developmental stage into both experimental design and clinical trial frameworks. Future studies will aim to dissect downstream TrkB‐independent signaling in females and explore clinical timing and dosing of FLX administration in translational models. Together, these findings lay the groundwork for mechanistically informed, age‐ and sex‐specific approaches to improve long‐term neurological outcomes in children who survive hypoxic‐ischemic brain injury.

## Disclosure

Part of this project was previously presented as a poster at the Society for Neuroscience Annual Meeting 2024, please see: https://www.sfn.org/-/media/SfN/Documents/NEW-SfN/Meetings/Neuroscience-2024/Abstracts-and-Sessions/ECPS---TPDA.pdf.

## Conflicts of Interest

The authors declare no conflicts of interest.

## Author Contributions

April Fineberg and Tanner McVey equally contributed to the project.

## Funding

This work was supported by the National Institutes of Health (1 R21 NS128784).

## Data Availability

The data that support the findings of this study are available from the corresponding author upon reasonable request.
